# The impact of perceived organizational justice on young nurses’ job performance: a chain mediating role of organizational climate and job embeddedness

**DOI:** 10.1186/s12912-024-01898-w

**Published:** 2024-04-07

**Authors:** Jiamei Song, Xindi Shi, Xiaojia Zheng, Guangli Lu, Chaoran Chen

**Affiliations:** 1 Institute of Nursing and Health, School of Nursing and Health, Henan University, 475004 Kaifeng, China; 2 Institute of Business Administration, School of Business, Henan University, 475004 Kaifeng, China

**Keywords:** Perceived organizational justice, Job performance, Organizational climate, Job embeddedness, Young nurse

## Abstract

**Background:**

The level of nurses’ job performance has always been of great concern, which not only represents the level of nursing service quality but is also closely related to patients’ treatment and prognosis. The aim of this study was to analyze the relationship between perceived organizational justice and job performance and to explore the mediating role of organizational climate and job embeddedness among young Chinese nurses.

**Methods:**

A cross-sectional survey of 1136 young nurses was conducted between March and May 2023 using convenience sampling. Data were collected using the Job Performance Scale, Organizational Justice Assessment Scale, Nursing Organizational Climate Scale, and Job Embeddedness Scale, and the resulting data were analyzed using SPSS 25.0 and AMOS 26.0.

**Results:**

There was a significant positive correlation between job performance and perceived organizational justice (*r* = 0.477, *p* < 0.01), organizational climate (*r* = 0.500, *p* < 0.01), and job embeddedness (*r* = 0.476, *p* < 0.01). Organizational climate and job embeddedness acted as chain mediators between perceived organizational justice and job performance. The total effect of perceived organizational justice on job performance (*β* = 0.513) consisted of a direct effect (*β* = 0.311) as well as an indirect effect (*β* = 0.202) mediated through organizational climate and job embeddedness, with the mediating effect accounting for 39.38% of the total effect.

**Conclusions:**

Organizational climate and job embeddedness play a chain mediating role between perceived organizational justice and job performance, so hospital managers should pay attention to the level of perceived organizational justice among young nurses, and develop a series of targeted measures to improve their job performance using organizational climate and job embeddedness as entry points.

## Introduction

The World Health Organization (WHO) predicts that the global elderly population will be twice as large in 2050 as it was in 2015 [1]. Therefore, comprehensive and even long-term person-centered care must be provided to the elderly population in order to maintain their health status and cope with global demographic changes [2]. Nurses, as professionals in the healthcare system, not only play an important role in the health education of patients [3, 4], but are also the main providers of long-term care services to patients [5, 6]. It can be argued that a sufficient number of nurses and a high level of quality of care are important for us to cope with demographic changes. However, the number of nurses is in a continuous shortage due to various reasons, such as the COVID-19 epidemic [7]. Therefore, how to solve the shortage of nurses has become a hot research topic among scholars. Previous quantitative and qualitative studies have shown that there is a significant negative correlation between nurses’ job performance and intention to leave [8, 9]. Wang et al.’s study also showed that job performance plays an important role in decreasing nurses’ intention to leave, that is, job nurses with higher performance tend to have lower turnover intention [10]. In addition, young nurses are more likely to be maladjusted to their nursing roles and thus have turnover intentions than older nurses [11]. Buerhaus et al.’s study also showed that the number of nurses under the age of 35 declined by 4% between February 2020 and June 2021 [12]. There is no doubt that the loss of young nurses will exacerbate the lack of nursing staff. Therefore, it is necessary to study the job performance of young nurses in order to reduce the turnover of nursing personnel and improve the quality of nursing services so as to better cope with demographic changes. (Note: According to China’s relevant policies and regulations, young nurses in this study refer to nurses between the ages of 18 and 35)

## Background

The concept of job performance is commonly found in management and refers to the scalable actions, behaviors, and outcomes of employees to achieve organizational goals [13]. Nurses’ job performance refers to the contributions and achievements of nurses in their clinical work using their knowledge and skills [14]. A high level of job performance not only implies a high quality of nursing care for nurses [15], but is also closely related to patient treatment and recovery [16]. Therefore, exploring the influencing factors of young nurses’ job performance is essential to improve the quality of nursing services and reduce the turnover of young nurses. In fact, a large number of studies, both nationally and internationally, have shown that, in addition to general demographic information such as age, gender, job title, and salary level [17–19], leadership style, resilience, job satisfaction, and even the use of social media have a significant impact on job performance [20–22].

As an important predictor of job performance, organizational justice refers to the extent to which an organization adheres to norms reflecting fairness and appropriateness in its decision-making process [23]. Previous studies have shown that employees with higher levels of perceived organizational justice are more likely to take the initiative to improve their job performance [24]. Yu’s study showed that there is a significant positive correlation between organizational justice and job performance among nurses [16]. Beatriz’s study also showed that the higher level of perceived organizational justice among employees is associated with higher level of job performance [25]. However, there are often unexplained discrepancies between the results of different studies [26], and most previous studies have focused on hotel employees [27], teachers [28], and physician populations [29], with young nurses’ research being more limited. Therefore, it is necessary to explore the mediating mechanisms by which perceived organizational justice affects job performance in order to gain a deeper understanding and improve the job performance of young nurses.

Organizational climate refers to a perception or feeling that members of an organization have about the characteristics of the environment in which the organization is located, which usually originates from the individual organization members’ psychological perceptions of the work environment in which they are located [30]. Huang pointed out that there is a positive correlation between employees’ organizational justice and organizational climate [31]. Su’s findings also showed that perceived organizational justice is an important influence on the organizational climate of young nurses [32]. In addition, social information processing theory suggests that organizational climate changes employees’ attitudes, behaviors, and beliefs at work, which in turn has an impact on employee performance [33]. Previous studies have shown a significant positive correlation between organizational climate and job performance [34, 35]. Hanife Tiryaki Sen and Aytolan Yildirim’s study also showed that a supportive organizational climate has a positive effect on the job performance of young nurses [36]. Therefore, this study proposes Hypothesis 1: Organizational climate is a mediating variable between perceived organizational justice and job performance of young nurses.

Job embeddedness refers to the closeness of the network of relationships formed between an individual and all work-related situations, and it encapsulates the organizationally relevant factors that make an employee remain on the job, which causes the employee to be attached or embedded in the job they are in [37]. Nurses’ job embeddedness, on the other hand, refers to all the positive factors that make nursing managers do their best to keep nursing staff in their nursing positions [38]. Lee’s study showed that organizational justice had a significant positive effect on the job embeddedness of clinical young nurses [39]. Kim’s study also found that young nurses with higher perceptions of organizational justice usually had higher job embeddedness compared to those with lower perceptions of organizational justice [40]. In addition, many previous studies have confirmed a significant positive correlation between job embeddedness and job performance among nursing staff [41–43]. Therefore, we proposed Hypothesis 2: Job embeddedness is a mediating variable between perceived organizational justice and job performance of young nurses.

Resource Conservation Theory suggests that a high degree of job embeddedness is a state of resourcefulness, and employees with a high degree of job embeddedness will not only show greater self-confidence and positive work attitudes in the face of work challenges, but will also improve their job performance by enhancing their critical skill reserves and problem solving abilities through various means [44, 45]. Similarly, Liu’s study found that young nurses with high levels of job embeddedness were more willing to improve their performance for the benefit of the organization [46]. Furthermore, Hashim’s study has confirmed a significant positive correlation between organizational climate and job embeddedness [47]. In light of this, we proposed Hypothesis 3: Job embeddedness is a mediating variable between organizational climate and young nurses’ job performance.

**Fig. 1:**
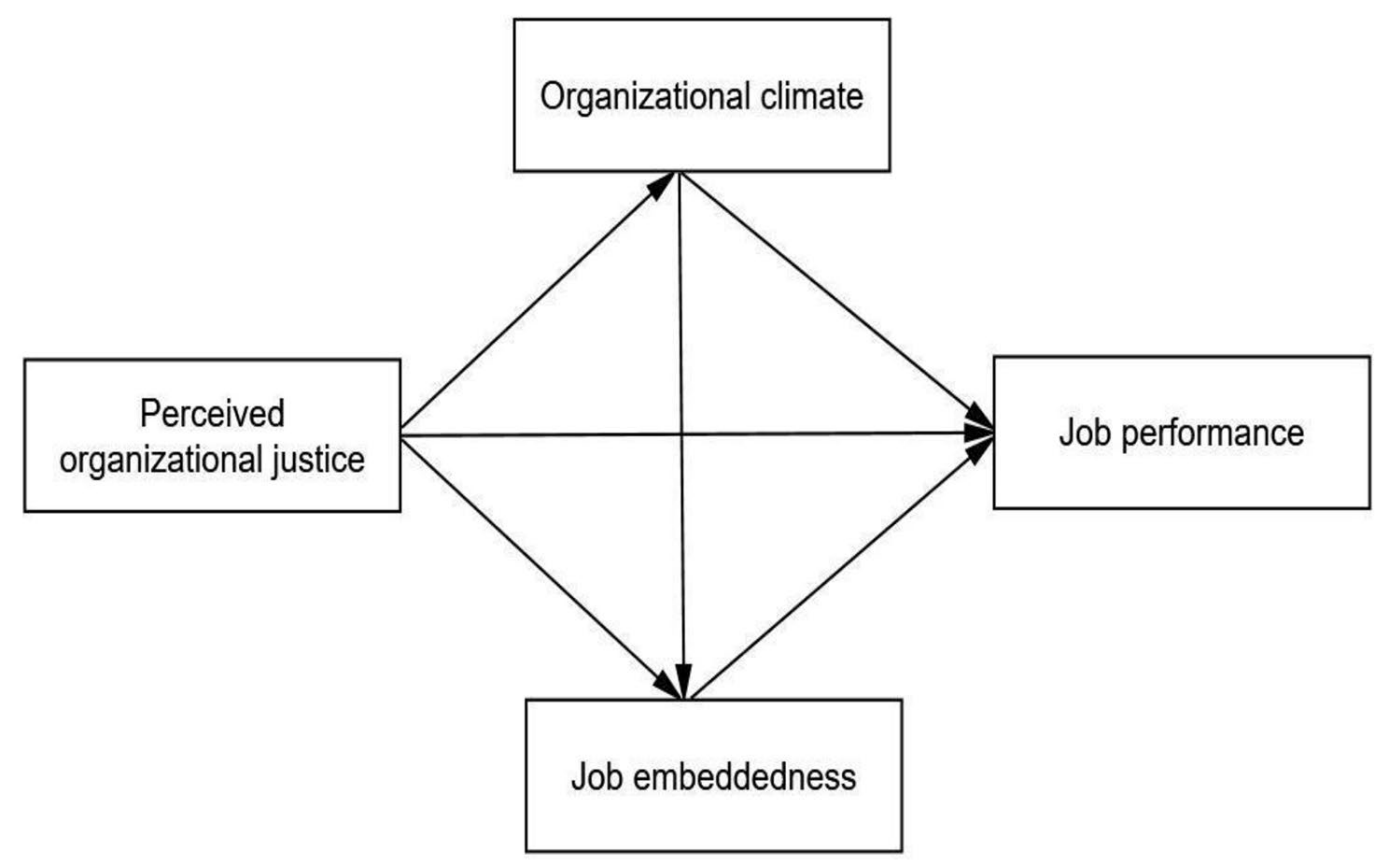
Conceptual Framework

In conclusion, although there have been studies on the relationship between perceived organizational justice and job performance, there is still a lack of research on the mechanisms between the two relationships, and very limited research on the young nurse population. Therefore, this study aimed to explore the effects of perceived organizational fairness on young nurses’ job performance and to test the mediating role of organizational climate and job embeddedness, with a view to providing ideas and guidance for research and interventions on job performance management for young nurses. The conceptual framework of this study is shown in Fig. 1.

## Methods

### Study design

This study conducted a cross-sectional survey from March to May 2023 to examine the relationship between perceived organizational justice, job embeddedness, organizational climate and job performance among young nurses.

### Participants

Participants in this study were drawn from nurses in six hospitals in Henan Province, China. Inclusion criteria were (a) aged between 18 and 35 years old; and (b) possessing a certificate of nursing practice. Exclusion criteria were (a) nurses in advanced training; (b) nurses on vacation during the survey period.

### Procedure

Convenience sampling method was used. The research team used a paper version of the questionnaire, which was collected face-to-face with the help of the hospital administrators. Prior to the start of the survey, participants were briefed about the purpose and significance of the study and assured that the data collected would be used for research purposes only. Participants were given the option to participate in the survey at their own discretion and to opt out at any time during the survey. We distributed 1,300 questionnaires, and 1,200 were eventually returned, with a return rate of 92.31%. After excluding 64 invalid questionnaires, 1136 valid questionnaires were recovered, with a valid recovery rate of 87.38%.

### Measures

#### Demographic characteristics

Demographic characteristics include age, gender, marital status, hospital grade (Tertiary, Secondary or below), educational background (Junior college or below, Bachelor’s, Master’s or above), whether the head nurse (Yes, No), professional title (Nurse, Senior nurse, Nurse-in-charge, Deputy chief nurse or above), And monthly income (< 4,000RMB, 4000∼7000, 7000∼10,000, > 10,000) etc.

#### Job performance

The Job Performance Scale developed by Van Scotter [48] and adapted by Yu De-Cheng (1996) was used to measure the job performance of young nurses. The scale consists of two dimensions, task performance (5 items) and contextual performance (6 items), with a total of 11 items. The Likert 5-point scale was used, with “1” indicating “not at all” and “5” indicating “fully”. The higher the score, the better the performance of young nurses. The Cronbach’s coefficient for this scale was 0.918 and in this study the Cronbach’s coefficient for this scale was 0.962.

#### Perceived organizational justice

The perceived organizational justice of young nurses was assessed with organizational justice scale. Which compiled by Colquitt [49] and translated by Yu Jingfen in 2022, included four dimensions: distributive justice (4 items), procedural justice (7 items), interpersonal justice (4 items) and informational justice (5 items), a total of 20 items. Likert 5 rating scale was used, ranging from “Completely disagree” to “Completely agree” from 1 to 5. The higher the score, the higher the perceived organizational justice of the young nurses. The Cronbach’s coefficient for this scale was 0.910 and in this study the Cronbach’s coefficient for this scale was 0.912.

#### Organizational climate

The Nursing Organizational Climate Scale developed by He [50] was used. The scale consists of 24 items in four dimensions: equitable support behavior (10 items), collegial behavior (5 items), interpersonal climate behavior (4 items), and intimate and aggressive climate behavior (5 items). The Likert 5-point scale was used, with scores ranging from 1 to 5 on a scale from “very non-conforming” to “very conforming”, with higher scores indicating better organizational climate. The Cronbach’s coefficient for this scale was 0.927 and in this study the Cronbach’s coefficient for this scale was 0.976.

#### Job embeddedness

The job embeddedness scale developed by Crossley [51] and translated by Mei Hua (2014) was used. The scale is unidimensional, with 7 items. A 5-point Likert scale was used, ranging from “1” to “5” for “not at all” to “perfectly”. The question “It is easy for me to leave this organization” was reverse scored. The higher the scale score, the higher the degree of job embeddedness of the young nurses. The Cronbach’s coefficient for this scale was 0.890 and in this study the Cronbach’s coefficient for this scale was 0.827.

### Statistical analysis

We used SPSS 25.0 and AMOS 26.0 for data analysis. First, descriptive statistics (frequencies, percentages, means, standard deviations, etc.) were used to measure participants’ demographic characteristics and job performance, perceived organizational justice, organizational climate, and job embeddedness. Second, we used Pearson correlation analysis to examine the relationship between the four variables of job performance, perceived organizational justice, organizational climate, and job embeddedness. Finally, the chained mediation model was tested using AMOS 26.0. Where perceived organizational justice is the independent variable and job performance is the dependent variable, both organizational climate and job embeddedness are mediating variables, and gender is a covariate in this model. In addition, to investigate the effect of perceived organizational justice on job performance, we performed bias-corrected percentile bootstrapping with a 95% confidence interval calculated from a bootstrap sample of 5000. *P* -values were two-tailed and *p* < 0.05 was considered statistically significant.

## Results

### The demographic characteristics of the participants

Of the 1136 participants, 73.70% were female, 26.30% were male, more than 50% were aged 18–24, and 77.90% of the young nurses were single. More than half of the young nurses have a bachelor’s degree (67.30%), most of them work in tertiary hospitals (71.40%), and most of them have worked for less than 5 years (84.25%). Fewer young nurses work in pediatrics (6.90%), emergency departments (7.70%), and intensive care units (5.5%). The title of the participants was mostly ‘nurse’ (65.50%), and very few young nurses (6.50%) were head nurses. More than half (55.1%) of the young nurses had a contract relationship with the hospital, and more than 80% of the young nurses had a monthly income of less than 7,000 yuan. See Table 1 for details.

**Table 1:** Demographic characteristics (*N* = 1136)

Variables		N	%
Gender	Male	299	26.30
	Female	837	73.70
Educational background	Junior college or below	234	20.6
	Bachelor’s	764	67.3
	Master’s or above	138	12.1
Years	18∼24	668	58.80
	25∼29	321	28.26
	30∼35	147	12.94
Marital status	Single	885	77.90
	Married	241	21.20
	Divorced or widowed	10	0.90
Hospital level	Tertiary	811	71.40
	Secondary or below	325	28.60
Head nurse?	Yes	74	6.50
	No	1062	93.50
Professional title	Nurse	744	65.50
	Senior nurse	257	22.60
	Nurse-in-charge	91	8.00
	Deputy chief nurse or above	44	3.90
Departments	Internal medicine	329	29.00
	Surgery	264	23.20
	Obstetrics and gynecology	119	10.50
	Pediatric	78	6.90
	Emergency	88	7.70
	Intensive care unit	63	5.50
	Others	195	17.20
Labor and personnel relations	Formal establishment	237	20.90
	Personnel agency	273	24.00
	Contract system	626	55.10
Years of working	< 1	474	41.73
	1–5	483	42.52
	6–10	133	11.71
	> 10	46	4.04
Monthly income	< 4000	467	41.11
	4000∼7000	448	39.44
	7000∼10,000	140	12.32
	> 10,000	81	7.13

### Pearson’s correlation analysis

The results of the mean, standard deviation and correlation coefficients of each variable in this study are shown in Table 2. The scores for job performance, perceived organizational justice, organizational climate, and job embeddedness were 41.12 ± 9.69, 72.53 ± 12.48, 91.48 ± 16.96, and 23.56 ± 4.77, respectively. In addition, Pearson’s correlation analysis found a positive correlation between job performance and perceived organizational justice (*r* = 0.477, *P* < 0.01), organizational climate (*r* = 0.500, *P* < 0.01) and job embeddedness (*r* = 0.476, *P* < 0.01). There was an association between perceived organizational justice and organizational climate (*r* = 0.488, *P* < 0.01) and job embeddedness (*r* = 0.425, *P* < 0.01). Furthermore, there was a significant positive correlation between organizational climate and work embeddedness (*r* = 0.699, *P* < 0.01).

**Table 2:** Correlation Analysis of job performance, perceived organizational justice, organizational climate, and job embeddedness (*N* = 1136)

	JP	POJ	OC	JE	Mean	Standard deviation
JP	1				41.12	9.69
POJ	0.477**	1			72.53	12.48
OC	0.500**	0.488**	1		91.48	16.96
JE	0.476**	0.425**	0.699**	1	23.56	4.77

JP, job performance; POJ, perceived organizational justice; OC, organizational climate; JE, job embeddedness. ** *P* < 0.01 (Two tailed).

### Mediating effect analysis

In this study, after using AMOS 26.0 to test for mediating effects and incorporating perceived organizational justice, job performance, organizational climate, and job embeddedness into the structural equation model analysis, the model fit indices were χ2/df = 3.583 (< 5.0), GFI = 0.974 (> 0.90), CFI = 0.989 (> 0.90), AGFI = 0.959 (> 0.90), TLI = 0.985 (> 0.90), IFI = 0.989 (> 0.90), RMSEA = 0.048 (< 0.08), indicating a good model fit.

**Fig. 2:**
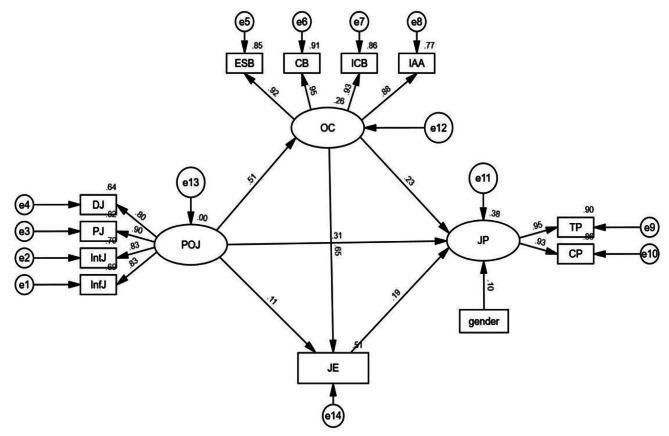
Path analysis diagram of perceived organizational justice, job performance, organizational climate, and job embeddedness. DJ, Distributive justice; PJ, Procedural justice; IntJ, Interpersonal justice; InfJ, Informational justice; POJ, perceived organizational justice; ESB, Equitable Support Behavior; CB, Collegial behavior; ICB, Interpersonal Climate Behavior; IAA, Intimate and Aggressive Atmosphere; OC, organizational climate; JP, job performance; TP, Task performance; CP, Contextual performance; JE, job embeddedness

As shown in Fig. 2, perceived organizational justice significantly and positively predicted job performance (*β* = 0.31, *P* < 0.001), organizational climate (*β* = 0.51, *P* < 0.001), and job embeddedness (*β* = 0.11, *P* < 0.001) of young nurses. Organizational climate was a significant positive predictor of job performance (*β* = 0.23, *P* < 0.001) and job embeddedness (*β* = 0.65, *P* < 0.001). Also, job embeddedness significantly and positively predicted job performance (*β* = 0.19, *P* < 0.001).

To verify the mediating effect of organizational climate and job embeddedness between perceived organizational justice and job performance, we used the Bootstrap method with percentile bias correction. The results showed a significant mediating effect of organizational climate and job embeddedness, with a total indirect effect value of 0.202, accounting for 39.38% of the total effect (0.513). Specifically, the mediating effects in this study included three paths. First, the coefficient of the indirect effect path of perceived organizational justice through organizational climate on job performance was 0.119 (Bootstrap 95% CI: 0.081, 0.161). Second, the path of indirect effect of perceived organizational justice on job performance through job embeddedness was 0.021 (Bootstrap 95% CI: 0.010, 0.035). Third, the indirect path of perceived organizational justice through organizational climate, job embeddedness on job performance was 0.062 (Bootstrap 95% CI: 0.036, 0.091). Details are shown in Table 3; Fig. 2.

**Table 3:** Total, direct, and indirect effects of perceived organizational justice on job performance (*N* = 1136)

Effects	Paths	Effect	Bootstrap SE	Bootstrapping 95% CI
Total effect	POJ → JP	0.513	0.029	0.453 to 0.568
Direct effect	POJ → JP	0.311	0.033	0.248 to 0.375
Indirect effect	POJ→OC→JP	0.119	0.021	0.081 to 0.161
	POJ→JE→JP	0.021	0.006	0.010 to 0.035
	POJ→OC→JE→JP	0.062	0.014	0.036 to 0.091
Total indirect effect	Total indirect effect	0.202	0.021	0.164 to 0.247

JP, job performance; POJ, perceived organizational justice; OC, organizational climate; JE, job embeddedness. SE, standard error; CI, confidence interval.

## Discussion

This study investigated the job performance of young nurses. The relationships between perceived organizational justice, organizational climate, job embeddedness, and job performance were explored, and a chain mediation model was constructed.

We found that there is a significant positive relationship between perceived organizational justice and young nurses’ job performance, and the higher the level of perceived organizational justice of young nurses, the higher the level of their job performance, which is consistent with the results of a previous study [16]. The result also reveals that we should create a fair and just working environment for young nurses, for example, hospital management staff should focus on communication with young nurses to achieve procedural fairness and distributive fairness, etc., in order to improve their sense of organizational fairness.

This study demonstrated that organizational climate is a mediating variable between young nurses’ perceived organizational justice and job performance, confirming Hypothesis 1. Young nurses with a high level of perceived organizational justice will have a more positive organizational climate, which will not only lead to greater satisfaction with their work but also to a greater willingness to comply with organizational rules and regulations [52, 53], resulting in higher motivation and work engagement [54, 55], which in turn leads to higher levels of job performance. Therefore, hospitals should consider more from the perspective of young nurses, increase the openness and transparency of management, and provide young nurses with channels to participate in the management of the organization so as to improve their sense of organizational justice. In addition, the head nurses of each department should strengthen the internal communication of the department, carry out diversified group activities to enhance the collective consciousness and organizational identity of young nurses, and create a positive organizational atmosphere, so as to achieve the purpose of improving the job performance of young nurses.

The results of this study confirmed that job embeddedness is a mediating variable between perceived organizational justice and young nurses’ job performance, validating Hypothesis 2. The level of job embeddedness of young nurses increases with the level of perceived organizational justice, which is consistent with the results of previous studies on other populations [56, 57]. And the higher the level of job embeddedness, the closer the young nurses are connected to their colleagues and the hospital [58, 59], which facilitates the enhancement of their work performance through teamwork. Therefore, hospital management should pay attention to the dual role of perceived organizational justice and job embeddedness, not only to create a fair and just working environment for young nurses but also to pay attention to their career development, provide young nurses with diversified training opportunities and promotion paths, link young nurses’ individual career development goals with the hospital’s development goals, and stimulate their motivation to deepen the young nurses’ job embedding level.

This study also found that job embeddedness is a mediating variable between organizational climate and job performance among young nurses, and Hypothesis 3 was confirmed. This may be due to the fact that a good organizational climate creates a sense of belonging and irreplaceability within young nurses towards the hospital [60–62], and these feelings deepen an individual’s job embeddedness, which enhances their intrinsic motivation to work and increases their motivation and work efficiency [63], which ultimately manifests itself in high levels of individual job performance. Therefore, it is necessary for hospital administrators to deepen young nurses’ connection with their colleagues and units, help them set career goals that are consistent with the direction of the hospital, and provide them with appropriate material and psychological rewards.

In addition, this study found that perceived organizational justice can have an impact on young nurses’ job performance through the chain-mediated effects of organizational climate and job embeddedness. This result suggests that young nurses with high levels of perceived organizational justice have more positive perceptions of the hospital and organizational climate [64] and are more inclined to develop stronger ties with their colleagues, leaders, and the hospital [39], which can contribute to enhancing their job satisfaction and work motivation [65] and improve job performance. On the contrary, young nurses who perceive a low level of organizational justice will be dissatisfied with the atmosphere of the work environment in which they live [66], which may make them resistant to establishing harmonious relationships with other nurses and the organization, which is detrimental to the improvement of job performance.

### Relevance of clinical practice

As the backbone of the nursing workforce, young nurses are prone to burnout when faced with heavy clinical nursing work, leading to a decline in the quality of nursing services, and in severe cases, even the intention to leave or leave the profession. However, the global demand for nursing services is increasing. Therefore, it is particularly important to improve the job performance of young nurses. In order to improve the job performance of young nurses, we give some suggestions with the results of this study.

First of all, for nursing managers, it is necessary to establish a scientific performance appraisal system to make a scientific and accurate assessment of the performance of young nurses. It is also necessary to develop good communication feedback mechanisms, strengthen communication with young nurses, and focus on their needs. On this basis, managers should strive to ensure that procedures are fair, involve young nurses in the processing process, and empower them to participate more in decision-making. For example, when developing a remuneration or promotion system, the views and recommendations of young nurses should be fully taken into account, and their sense of involvement in the development of procedures should be enhanced, thereby enhancing their perception of organizational justice.

Second, hospitals can provide a supportive working atmosphere for young nurses by improving the working environment and providing employee benefits. Each department should also hold regular group activities to cultivate the spirit of cooperation among young nurses, so that they can obtain positive emotional experiences from group cooperation, which is conducive to making young nurses perceive a more harmonious and positive organizational atmosphere. In addition, hospitals should pay attention to the cultivation of young nurses’ professional identity, regularly carry out relevant courses and training, and also recognize and encourage the performance of young nurses, help them to make good career planning, so that young nurses can make clear the path of their career development.

Finally, the whole society should give more respect and recognition to nurses and the nursing profession, so as to deepen the job embedded level of young nurses. In a word, in actual clinical management, leaders should assess the perceived organizational justice, organizational climate, and work embeddedness of young nurses in a timely manner, and develop targeted intervention programs to improve the performance of young nurses.

### Limitations

This study has some limitations. Firstly, the subjects of this study are all young nurses in Henan Province, so the results may not be generalizable, and in the future, we can select young nurses from different regions to conduct more studies. Secondly, we only used the method of self-filling questionnaires to collect data, and the results are subjective, so there may be common method bias. In the future, we can try to use different data collection methods to reduce bias and make the research results closer to the real situation. Finally, this study only reveals the effect of perceived organizational justice on job performance and the mediating role of organizational climate and job embeddedness, and in the future, the mediating mechanism of other variables between the two can be studied in depth, in order to further enrich the model of perceived organizational justice affecting job performance.

## Conclusion

This study investigated perceived organizational justice, organizational climate, job embeddedness, and job performance of selected young nurses in Henan Province. In order to explore the process by which perceived organizational justice affects young nurses’ job performance, a chain mediation model was developed. The results of the study showed that organizational climate and job embeddedness played the role of chain mediators between perceived organizational justice and job performance of young nurses, and the model ‘perceived organizational justice→organizational climate→job embeddedness→job performance of young nurses’ was verified. Therefore, hospital administrators should take targeted interventions to improve young nurses’ perceived organizational justice, organizational climate, and job embeddedness to improve young nurses’ job performance.

## Data Availability

The datasets generated and analysed during the current study are not publicly available but are available from the corresponding author on reasonable request.
